# Comparative study of anxiety and depression following maxillofacial and orthopedic injuries. Study from a Nigerian University Teaching Hospital

**DOI:** 10.1002/cre2.90

**Published:** 2017-11-17

**Authors:** Ramat Oyebunmi Braimah, Dominic Ignatius Ukpong, Kizito Chioma Ndukwe, Akinyele Lawrence Akinyoola

**Affiliations:** ^1^ Department of Dental and Maxillofacial Surgery Usmanu Danfodio University Teaching Hospital Sokoto Nigeria; ^2^ Department of Mental Health Obafemi Awolowo University /Obafemi Awolowo University Teaching Hospitals Complex, Ile‐Ife Osun State University of Nigeria Nigeria; ^3^ Department of Oral & Maxillofacial Surgery and Oral Pathology Obafemi Awolowo University Teaching Hospitals Complex Osun State Nigeria; ^4^ Department of Orthopaedic Surgery and Traumatology Obafemi Awolowo University/Obafemi Awolowo University Teaching Hospitals Complex Osun State Nigeria

**Keywords:** anxiety, depression, long bone, maxillofacial

## Abstract

This study hopes to compare levels of anxiety and depression in the maxillofacial and orthopedic injured patients over a period of 12 weeks. This was a prospective, repeated measure design. A total of 160 participants (80 with maxillofacial and 80 with long bone fractures) had repeated review follow‐ups within 1 week of arrival in the hospital (Time 1), 4–8 weeks after initial contact (Time 2) and 10–12 weeks thereafter (Time 3), using hospital anxiety and depression scale questionnaire. Road traffic accident remained the main cause of injury in both groups of subjects. The Hospital anxiety and Depression scale detected 42 (52.5%) cases of depression at baseline, 36 (47.4%) cases at Time 2, and 14 (18.4%) cases at Time 3 in the maxillofacial injured group. In the long bone fracture subjects, 47 (58.8%) cases were depressed at baseline, 23(33.3%) cases at Time 2, and only 5 (7.2%) cases at Time 3. Both groups showed reduction in depression levels with time. Fifty‐six (70.0%) had anxiety at baseline, 32 (42.1%) at Time 2, and only 9 (11.8%) had anxiety at Time 3 in the maxillofacial fracture group, whereas in the long bone fracture group, 69 (86.3%) subjects were anxious at baseline, 32 (46.4%) at Time 2, and 22 (31.9%) at Time 3. There were significant differences in depression and anxiety level in both the maxillofacial and the long bone fracture subjects at baseline (Time 1), Time 2(4–8 weeks) and Time 3(10–12 weeks).

## 1 INTRODUCTION

The psychological needs of patients with acquired facial trauma are unique and very important. It has been noted that patients with orofacial trauma were more likely to report symptoms of depression, anxiety, and hostility when compared to a matched normal control group for a period of up to 1 year post trauma (Bisson, Sheperd, & Dhutia, 1997). Many studies have reported that 10–70% patients based on various factors may experience symptoms of depression and anxiety after facial trauma (Bisson et al., 1997). This may be coupled with the fact that patients with orofacial trauma have psychosocial problems such as unemployment, lower education level, and poor social support. (Levine, Degutis, Pruzinsky, Shin, & Persing, 2005) The symptoms of depression and anxiety in many cases may be subthreshold and may not meet the full diagnostic criteria of a psychiatric disorder. This may often lead to diagnostic dilemmas, poor treatment of the problem, and poor intervention. Reactions such as normative sadness, grief over the losses they have experienced, reactions to medications they may be taking, and fatigue that results from treatment may be confused to being a depressive disorder or episode. Depression places the patient at increased risk for committing suicide, poor compliance with treatment, and poor rehabilitation outcome. This in turn will affect the quality of life and recovery from the facial trauma (Cuijpers & Smit, 2004; Meningaud, Benadiba, Servant, Bertrand, & Pelicier, 2003).

Depression and anxiety associated with facial trauma are often coupled with worries regarding recovery and length of the treatment process (Enqvist, Von konow, & Bystedt, 1995). Facial trauma leads to disfigurement that also affects the social image of the patient (McGrouther, 1997). Patients may express unhappiness regarding facial appearance after facial trauma and this may lead to social withdrawal and isolation. They may feel inferior to others in social presentation and may often feel a stigma associated with facial disfigurement (Newell, 2000).

Often the injuries are due to family fights and interpersonal assault, while a third of the patients have a previous history of a facial traumatic injury (Le, Dierks, Ueeck, Homer, & Potter, 2001). The recovery process after facial trauma is often lengthy and multiple surgeries with a multidisciplinary postoperative rehabilitation process may be needed. This may add to the frustration of the patient (Van Swearingen, 2008). Injuries to key areas of the face such as the eyes, ears, and dental injuries often increase vulnerability to stress and impede recovery (Shaikh & Worall, 2002) Significant difficulties in returning to premorbid levels of occupational functioning have been noted in these cases (Thompson & Kent, 2001)

Facial trauma patients also report higher rates of somatoform symptoms, substance abuse, post‐traumatic stress disorder symptoms, body image issues, stigmatization, lower quality of life, and lower overall satisfaction with life (Shepherd, 1992). Also, facial trauma patients report problems in marital, occupational, and social functioning (Tebble, Thomas, & Price, 2004). They also reported no correlation between the degree of disfigurement and the type, extent and severity of psychological response (Sen, Ross, & Rogers, 2001)

Similarly, several studies of patients with orthopedic trauma have focused on measures of functional recovery, complications, mortality, and costs (Adams, Davis, & Alexander, 2003; Moed, Yu, & Gruson, 2003; Pollak, McCarthy, & Bess, 2003; Richmond, Aharonoff, & Zuckerman, 2003). Less attention has been focused on patient psychological status (anxiety and depression) and quality of life following orthopedic trauma—a common source of patient complaints and a clinically relevant outcome (Rusch, 1998). Psychological symptoms following musculoskeletal trauma from various studies have ranged from 6.5% to 51.0% (Dijkstra, Groothoff, & Ten Duis, 2003; Mason, Wardrope, & Turpin, 2002; McCarthy, MacKenzie, & Edwin, 2003; Rusch, 1998; Starr, Smith, & Frawley, 2004). Despite overwhelming evidence that noninjury‐related factors have an important role in recovery from trauma, specific variables associated with clinical outcomes are poorly understood (MacDermid, Donner, & Richards, 2002; Mock, MacKenzie, & Jurkovich, 2000; Suter, 2002). This lack of knowledge complicates efforts to improve the care of orthopedic trauma patients.

Studies on this aspect of trauma has been carried out in developed countries; however, such studies are rare in underdeveloped countries especially in sub‐Saharan Africa. Therefore, the aim of this study is to compare levels of anxiety and depression in patients that have suffered maxillofacial and orthopedic injuries.

## 2 MATERIALS AND METHODS

### 2.1 Study settings

The subjects were consecutive patients who presented at the Accident and Emergency unit, Oral/Maxillofacial Surgery and Orthopaedic & Traumatology Units of the Obafemi Awolowo University Teaching Hospital Complex, Ile‐Ife, Nigeria. Subjects were recruited over a period of 12 months from February 2012 to January 2013, after approval from the hospital's Ethics and Research Committee.

Participants were patients with maxillofacial and orthopedic fracture who gave informed consent for the study. The inclusion criteria were age 18 years and above and Glasgow Coma Scale of 12 and above on admission. Patients with both maxillofacial and orthopedic injuries were excluded from the study. Baseline interview was conducted within 1 week of arrival in the hospital (Time 1). Follow up interviews were conducted at intervals of 4–8 weeks after initial contact (Time 2) and 10–12 1weeks thereafter (Time 3).

### 2.2 Socio‐demographic and clinical data

The data were obtained using a specially prepared questionnaire. Documentation at baseline included age, gender, educational status, employment status, and marital status. The clinical information includes etiology of injury, site of injury, type of injury, and whether treatment was operative or conservative and duration of hospital admission.

### 2.3 Hospital anxiety and depression scale (HADS) instrument

This is a 14‐item self‐reporting instrument with anxiety and depression subscales (Zigmond & Snaith, 1983). Each item is rated on a 4‐point scale, with each subscale having a range of 0–21. The HADS has been validated in Nigerian hospital and community samples (Abiodun, 1994). The recommended cut‐off score of 7 for this locality was used for this study (Abiodun, 1994).

### 2.4 Statistical analysis

Data were analyzed with SPSS version 16 (SPSS 16 Inc., Chicago, IL, USA). Results were calculated as frequencies (%), means, and standard deviations (SD) for normally distributed variables. Independent Samples *T* test was used to compare mean score values for the HADS and between group differences. Probabilities of less than 0.05 were accepted as significant.

## 3 RESULTS

### 3.1 Socio‐demographics

The study population consisted of 160 participants (80 with maxillofacial fractures and 80 with long bone fractures). There were 122 (76.3%) male and 38 (23.7%) female participants. The mean age of the sample was 33.2±12.5, range 18–70 years. However, the maxillofacial fracture group was younger than the long bone fracture group with mean age of 30.9±11.3 and 37.6±12.8 respectively. There was statistically significant difference when the age was compared for the two groups *p* = .001.

Road traffic accidents were responsible for a sizeable proportion of injuries in both groups, 68 (85%) in the maxillofacial fractured and 73 (91.3%) in the long bone fracture group.

The socio‐demographic characteristics of the study population are shown in Tables 1a.

**Table 1a:** Socio‐demographic characteristics

	Maxillofacial fracture	Long bone fracture	Total	*p* value
Sex				
Male	64 (80.0%)	58 (72.5%)	122 (76.3%)	0.265
Female	16 (20.0%)	22 (27.5%)	38 (23.7%)	
Age				
Young adult (18–35 years)	60(75.0%)	44 (55.0%)	104 (65.0%)	0.028
Middle age(36–44 years)	10 (12.5%)	16 (20.0%)	26 (16.3%)	
Elderly(45–70 years)	10 (12.5%)	20 (25.0%)	30 (18.7%)	
Marital status				
Married	47 (58.8%)	23 (28.8%)	70 (43.8%)	0.001
Single	33 (41.2%)	56 (70.0%)	89 (56.6%)	
Divorced	0 (0.0%)	1 (1.2%)	1 (0.6%)	
Education				
No education	2 (2.5%)	8 (10.0%)	10 (6.3%)	0.105
Primary	13 (16.2%)	21 (26.3%)	34 (21.3%)	
Secondary	41 (51.3%)	30 (37.5%)	71 (44.4%)	
Tertiary	24 (30%)	21 (26.2%)	45 (28%)	
Occupation				
Unemployed	21 (26.3%)	8 (10.0%)	29 (18.1%)	0.020
Unskilled	30 (37.5%)	43 (53.8%)	73 (45.6%)	
Skilled	14 (17.5%)	16 (20.0%)	30 (18.8%)	
Professional	12 (15.0%)	8 (10.0%)	20 (12.5%)	
aOthers	3 (3.7%)	5 (6.2%)	8 (5.0%)	
Etiology				
Assault	6 (7.5%)	0 (0.0%)	6 (3.8%)	0.044
Road traffic accident	68 (85.0%)	73 (91.5%)	141 (88.1%)	
Others (fall and occupational injury)	6 (7.5%)	7 (8.7%)	13 (8.1%)	

a others (students, youth corpers)

Only 21 patients where admitted in the maxillofacial fractured group and most of them were discharged home within 1 week of hospital stay (16 [76.2%]), whereas 71 patients were admitted in the long bone fracture group and majority stayed over 12 weeks on admission (59 [83.1%]) as shown in Table 1b.

**Table 1b:** Distribution of duration of hospital stay and injury

Duration of hospital stay	Maxillofacial fracture (*n* = 21)	Long bone fracture (*n* = 71)
< 1 week	16 (76.2%)	3 (4.2%)
4–8 weeks	5 (23.8%)	7 (9.9%)
10–12 weeks	—	2 (2.8%)
> 12 weeks	—	59 (83.1%)
Total	21 (100%)	71 (100%)

*p* = 0.000

### 3.2 Depression

The HADS detected 42 (52.5%) cases of depression at baseline, 36 (47.4%) cases at Time 2, and 14 (18.4%) cases at Time 3 in the maxillofacial fracture group. In the long bone fracture subjects, 47 (58.8%) cases were depressed at baseline, 23(33.3%) cases at Time 2, and only 5 (7.2%) cases at Time 3 (These are subjects that scored above the cut‐off point of 7 on the Depression scale of the HADS). Both groups showed reduction in depression levels with time (Table 2).

**Table 2:** Change in mean HADS depression scores (M±SD) with time between groups

	Maxillofacial fracture	Long bone fracture	*p* value
Time 1 (within 1 week of injury)	(*n* = 80)	(*n* = 80)	
	8.4 (3.4)	8.3 (4.6)	0.664
	42 (52.5%) a	47 (58.8%) a	0.018
Time 2 (4–8 weeks)	(*n* = 76)	(*n* = 69)	
	7.4 (2.5)	6.6 (2.2)	0.248
	36 (47.4%) a	23 (33.3% ) a	0.189
Time 3 (10–12 weeks)	(*n* = 76)	(*n* = 69)	
	6.4 (1.7)	5.7 (1.6)	0.805
	14 (18.4%) a	5 (7.2%) a	0.132

a Proportion of subjects with high depression score

### 3.3 Anxiety

Fifty‐six (70.0%) had anxiety at baseline, 32 (42.1%) at Time 2, and only 9 (11.8%) had anxiety at Time 3 in the maxillofacial fracture group, whereas in the long bone fracture group 69 (86.3%) subjects were anxious at baseline, 32 (46.4%) at Time 2, and 22 (31.9%) at Time 3. Both groups of participants showed reduction in anxiety levels with time, but there were no significant differences when both groups were compared at Times 1, 2, and 3. (Table 3)

**Table 3:** Change in mean HADS anxiety scores (M±SD) with time between groups

	Maxillofacial fracture	Long bone fracture	*p* value
Time 1 (within 1 week of injury)	(*n* = 80)	(*n* = 80)	
	10.8 (3.3)	11.6 (3.7)	0.719
	56 (70.0%) a	69 (86.3%) a	0.491
Time 2 (4–8 weeks)	(*n* = 76)	(*n* = 69)	
	6.5 (3.2)	7.2 (2.9)	0.381
	32 (42.1%) a	32 (46.4%) a	0.189
Time 3 (10–12 weeks)	(*n* = 76)	(*n* = 69)	
	3.9 (3.1)	5.7 (3.7)	0.262
	9 (11.8%) a	22 (31.9%) a	0.132

a Proportion of subjects with high anxiety scores

## 4 DISCUSSION

The management of maxillofacial and orthopedic trauma is largely driven by the obvious clinical manifestations of the physical injury, while the less evident psychosocial sequelae are rarely considered (Lento et al., 2004; Remizov & Elena, 2008). Documented possible symptoms of these psychological sequelae following facial trauma include increase in levels of depression, anxiety, phobic anxiety, and obsessive compulsive tendencies (Lento et al., 2004). The appearance and “attractiveness” of a person to other people is partly contributed by the person's face. As a result of maxillofacial trauma, the patient may suffer facial disfigurement. Similarly, following orthopedic injuries, there may be loss of mobility in the joints that makes patient more dependent on others that affects their quality of life (Remizov & Elena, 2008).

### 4.1 Depression

This study has shown high levels of depression in both groups of subjects. The findings are similar to those of previous researches investigating psychosocial complications of traumatic injury (Bisson et al., 1997; Hull et al., 2003; McCarthy et al., 2003; Ukpong, Ugboko, Ndukwe, & Gbolahan, 2007). A similar findings in a previous Nigerian study reported that 41.2%, 47.1%, and 21.7% were cases of depression at Times 1(within 10 days of injury), 2(6–8 weeks after injury), and 3 (10–12 weeks after injury), respectively (Ukpong et al., 2007). This similarity was because both studies were carried out in similar study population and environment. The cause of injury was also similar.

When the two groups of subjects were compared, facial injured patients were more depressed at Times 2 and 3 (47.4% and 18.4%, respectively) than long bone fracture subjects (33.3% and 7.2%, respectively). This pattern is probably because 52 (65%) of the facial injured subjects had associated facial soft tissue injuries (Table 4) with the accompanying permanent scarring that could not be concealed. This permanent scarring may change their appearance and identity leading to social withdrawal and loss of self‐esteem (Thompson & Kent, 2001). Also, scarring may be the cause of ongoing depression and be a constant reminder of the accident or act of violence in which the injury was sustained (Shepherd, 1990).

**Table 4:** Distribution of types of maxillofacial soft tissue injuries

Type of soft tissue injury	(%)
Abrasion	3 (5.8)
Contusion	2 (3.8)
Laceration	16 (30.7)
Avulsion	1 (1.9)
Combination (abrasion, laceration, and avulsion)	30 (57.8)
Total	52 (100)

Although, the depression levels were reducing over the review periods, it did not completely abate after the review period. Lento et al. (2004) have reported similar findings whereby despite the reduction in symptoms of psychological distress over time, patients in the injury group continued to report more psychologic problems than the comparison cohort. Other studies (Lento et al., 2004; Whetsell, Patterson, Young, et al., 1989) have opined that post‐traumatic symptomatology may be an extension of preexisting psychosocial pathology and these patients may be poorly equipped psychologically to withstand the stresses of the injury and recovery. Preexisting psychological status of individuals in underdeveloped countries is not a routine exercise, therefore background psychological status of our patients were not known. Despite this limitation in sub‐Saharan Africans, this study has shown that there is a psychological component to trauma patients that must be addressed as anxiety.

For the maxillofacial fracture group, 70.0% were cases of anxiety at baseline compared to 86.3% in the long bone fracture group; whereas for Time 2, 42.1% were cases of anxiety in the maxillofacial fracture group compared to 46.4% in the long bone fracture group. At Time 3, 11.8% of the maxillofacial injury groups were anxious as compared to 31.9% in the long bone fracture group. However, when the mean anxiety scores for Times 1, 2, and 3 where compared for the two groups of subjects, no statistically significant value were found. Although, no statistically significant difference was observed, the long bone fractured subjects showed higher anxiety levels at Times 1, 2, and 3. One can speculate that the fear of inability to walk normally or risk of amputation and length of hospital admission could be responsible for this difference. From this study, mean hospital stay was 7.3(±4.1) days in the maxillofacial fracture and 38.6(±26.5) days in the long bone fracture subjects (Table 1b). In addition, there may be joint stiffness as a result of prolonged immobilization leading to reduced routine and specific activities. This finding has been previously reported in the literature following orthopedic injuries were they found out that one in every five patients met the criteria for psychological illness (Aggarwal, Kohli, Nagi, & Kumar, 2004).

Both groups of subjects were anxious from this study. This is comparable to previous reports of high rate of psychosocial complication following maxillofacial trauma (Bisson et al., 1997; Hull et al., 2003), and long bone fractures (Bhandari et al., 2008; Dijkstra et al., 2003; McCarthy et al., 2003; Remizov & Elena, 2008). This present findings contrast those of previous study in this environment (Ukpong et al., 2007) where investigators reported that 11.8% of those with maxillofacial injuries experienced high anxiety levels immediately after injury, 3.0% at 4–8 weeks and 13.0% at 10–12 weeks follow‐up periods. Although both studies were conducted in a similar environment, the reason for this difference could not be explained; however, the higher attrition rate could be responsible. Extensive literature search yielded no published data comparing psychological distress between these two groups of patients, however, this study could serve as the baseline reference in sub‐Saharan Africa. In addition, our findings echoed the need for attending surgeons and other healthcare providers to look out for these psychosocial distress in addition to physical injuries sustained by the patients. Furthermore, trauma care givers should be sensitized and trained in providing brief psychologic assessments.

## 5 CONCLUSION

Our data have highlighted the substantial, and largely unmet, mental health and service needs among maxillofacial and orthopedic injured subjects. There were significant differences in depression and anxiety levels in both the maxillofacial and the orthopedic injured subjects at baseline (Time 1), Time 2(4–8 weeks), and Time 3(10–12 weeks) with the maxillofacial injured recording higher levels of depression and the long bone fracture recording higher levels of anxiety. These findings have shown that management of these group of patients should be multidisciplinary involving psychologists, psychiatrist, social health workers, and the attending surgeons.

## CONFLICTS OF INTEREST

None declared.

## STATEMENT FROM AUTHORS

This manuscript has been read and approved by all the authors, and the requirements for authorship as stated earlier in this document have been met. Each author also believes that the manuscript represents honest work and has not been submitted, accepted, or published in any other journal.
